# Depression, Anxiety, Impulsivity, and Cognitive Performance in Elderly Psychiatric Patients

**DOI:** 10.1192/j.eurpsy.2026.11592

**Published:** 2026-07-31

**Authors:** N. Smaoui, M. Barkallah, R. Feki, M. Maalej, S. Omri, I. Gassara, N. Charfi, L. Zouari

**Affiliations:** Psychiatry Department “C”, Hedi Chaker Hospital, Sfax, Tunisia

## Abstract

**Introduction:**

Depression and anxiety are common in elderly psychiatric patients and are often associated with cognitive decline and behavioral dysregulation. Understanding the relationships between mood symptoms, impulsivity, and cognitive performance is crucial for tailored interventions in this vulnerable population.

**Objectives:**

We aimed to identify the associations between depressive and anxiety symptoms, impulsivity traits, and cognitive functioning in older adults receiving psychiatric care.

**Methods:**

A cross-sectional study was conducted among patients aged 65 years and older attending outpatient psychiatric consultations. Psychometric assessments included the Hospital Anxiety and Depression Scale for anxiety (HADS-A) and depression (HADS-D), the Barratt Impulsiveness Scale – 11th version (BIS-11) for impulsivity, and the Mini-Mental State Examination (MMSE) for cognitive function. Correlations between mood symptoms, impulsivity, and cognitive performance were analyzed.

**Results:**

Seventy-three patients were included (male/female ratio = 1.08). The mean MMSE score was 22.63 (range: 11–30), indicating cognitive impairment in 42 patients (58%).

The median HADS-D score was 10/21 (IQR = [6–14]; range: 2–18), with depressive symptoms (HADS-D ≥ 11) observed in 39 patients (53%).

The median HADS-A score was 9/21 (IQR = [6.5–12]; range: 3–19), with anxiety symptoms (HADS-A ≥ 11) present in 49 patients (67%). The median BIS-11 score was 84 (IQR = [65.5–97.5]), with values ranging from 41 to 112.

Significant positive correlations were observed between depression scores (HADS-D) and impulsivity (BIS-11) (p = 0.005), as well as between anxiety scores (HADS-A) and impulsivity (BIS-11) (p < 0.01), indicating that higher levels of mood symptoms were associated with increased impulsivity. Additionally, depression (HADS-D) and anxiety (HADS-A) were negatively correlated with cognitive performance (MMSE) (p = 0.005 and p < 0.01, respectively), suggesting that higher mood symptom severity was linked to greater cognitive impairment.

**Conclusions:**

In elderly psychiatric patients, depressive and anxious symptoms are associated with increased impulsivity and reduced cognitive function. These findings highlight the need for comprehensive assessment and integrated management of mood, behavior, and cognition in this population to improve clinical outcomes.

**Disclosure of Interest:**

None Declared

